# Overview of Ecology and Aspects of Antibiotic Resistance in *Campylobacter* spp. Isolated from Free-Grazing Chicken Tissues in Rural Households

**DOI:** 10.3390/microorganisms12020368

**Published:** 2024-02-10

**Authors:** Argyrios Dermatas, Georgios Rozos, Konstantinos Zaralis, Aikaterini Dadamogia, Konstantina Fotou, Eugenia Bezirtzoglou, Konstantoula Akrida-Demertzi, Panagiotis Demertzis, Chrysoula (Chrysa) Voidarou

**Affiliations:** 1 Food Chemistry Laboratory, Section of Industrial and Food Chemistry, Department of Chemistry, University of Ioannina, 45110 Ioannina, Greece; argydermatas@yahoo.gr (A.D.); kakrida@uoi.gr (K.A.-D.); pdemertz@uoi.gr (P.D.); 2 Laboratory of Animal Health, Food Hygiene and Quality, Department of Agriculture, University of Ioannina, 47132 Arta, Greece; clevervet@hotmail.com (G.R.); kdadamogia@gmail.com (A.D.); kfotou@uoi.gr (K.F.); 3 Department of Agriculture, School of Agricultural Sciences, University of Western Macedonia, 53100 Florina, Greece; kzaralis@uowm.gr; 4 Laboratory of Hygiene and Environmental Protection, Faculty of Medicine, Democritus University of Thrace, 68100 Alexandroupolis, Greece

**Keywords:** Campylobacter, ecology, antibiotic resistance, free-grazing chicken, *bla*_OxA-61_, *tet*(*O*), *tet*(*A*), *cmeA*, *cmeB*, *cmeC*, *gyrA* (Thr-86-Ile mutation)

## Abstract

Rural households all over the world rear backyard chicken mainly for their own consumption and, to a lesser extent, for barter trade. These chickens represent a staple dish with numerous culinary variations and a cheap source of protein. Although some *Campylobacter* species, and particularly *Campylobacter jejuni* and *Campylobacter coli*, have been associated with industrial poultry carcasses, studies concerning the ecology of this genus in rural households do not exist. To assess the prevalence of *Campylobacter* species in the tissues of backyard chickens, samples were collected from birds *Gallus domesticus* bred in households in the rural area of Epirus (Greece), and *Campylobacter* strains were isolated by quantitative methods at 37 °C and 42 °C. In total, 256 strains were identified, belonging to 17 *Campylobacter* species, with *C. jejuni* and *C. coli* being the most prevalent. From the four ecological parameters studied (size of the flock, presence of small ruminants in the same household, presence of other poultry species in the same household, and feeding leftovers of the household), the size of the flock and the presence of small ruminants and/or pigs in the same household mostly affected the distribution of these strains. To study the phenotypical resistance against 14 antibiotics, 215 strains were selected. The results showed a high prevalence of multidrug-resistance (MDR) strains extending to all classes of antibiotics. Further genome analysis revealed the presence of genes coding resistance (*bla*_OxA-61_, *tet*(*O*), *tet*(*A*) *cmeA*, *cmeB*, *cmeC*, and *gyrA* (Thr-86-Ile mutation)), with the efflux pump CmeABC being the most prevalent. All antimicrobial resistance-encoded genes co-circulated, except for *bla*_OXA-61_, which moved independently. The minimum inhibitory concentration (MIC) values of two out of three antibiotics (representing different classes) were reduced when the strains tested were exposed to carbonyl cyanide 3-chlorophenylhydrazone (CCCP), a known efflux pump inhibitor. The same result was obtained with the addition of CCCP to the MIC values of bile salts. These results lead to the conclusion that *Campylobacter* species are present in an impressive diversity in backyard chicken tissues and that they exert a significant resistance to antibiotics, raising a potential danger for public health.

## 1. Introduction

In 2021, campylobacteriosis, the infectious diseases caused by members of the bacterial genus *Campylobacter*, were the most reported zoonosis in the European Union (EU), followed by salmonellosis, with 127,840 cases—a 2.1% increase in the EU notification rate compared with 2020 [1]. Because many more cases go undiagnosed, it is estimated that campylobacteriosis affects over 1.3 million people every year [2]. *Campylobacter* species, and particularly *Campylobacter jejuni* and *Campylobacter coli* as major contributors to foodborne and waterborne infections, are among the leading causes of bacterial gastroenteritis on a global scale [3]. The overwhelming proportion of these statistics covers cases associated with broiler carcass meat [4,5,6]. What is usually overlooked is the backyard chicken, which is universally expanded in all rural populations and on every continent [7,8]. It is a cheap, easy-to-grow, easy-to-handle, and easy-to-process source of protein. A staple food presented in a culinary variety. According to the United Nations (UN), the rural population reached 5% of the global population in 2018, and it is estimated that this percentage will decline to 32% by 2050 [9]. The majority of these people rear chicken and other species in their backyard. Yet, very few, if any, papers have been published on the ecology of this genus in backyard chicken. One reason is that most human Campylobacteriosis cases are self-limiting, solved on their own without the need for specific antibacterial treatment, and the infection typically runs its course without medical intervention [10,11]. Another reason is that *Campylobacter* infections rarely cause a clinical disease in chickens [12]. A third reason could be that the broiler industry and the associated logistics (transport and storage of the carcasses) offer a controlled environment for research without all the multitude of conditions and situations of rural households. Yet, it is this multitude that may create excellent ecological niches for various species of the *Campylobacter* genus to grow and thrive (or not).

It follows that this ecological multitude will not only influence the prevalence of various *Campylobacter* species but also impact their resistance to antibiotics. Contact with other bacteria may lead to the horizontal transfer of genetic determinants of resistance. In both human and veterinary clinical practice, *Campylobacter* species, especially *C*. *jejuni*, have exhibited an increasing trend of resistance to various classes of antibiotics, with fluoroquinolones and macrolides being particularly noteworthy [11,13,14]. Furthermore, an expanding body of research reports the emergence of multiple drug-resistant *Campylobacter* strains, presenting challenges to the effective treatment of human *Campylobacter* infections [14,15,16,17,18].

The ability of *Campylobacter* to undergo horizontal gene transfer, facilitated by mobile genetic elements like plasmids, allows for the exchange of genetic material, including antibiotic resistance genes, between different strains. This contributes to the evolution and dissemination of resistance and facilitates the spread of resistance among bacterial populations [14,19]. The use of antibiotics in poultry farming, including in free-range systems, can exert selective pressure, favoring the survival and proliferation of antibiotic-resistant strains. The emergence of antibiotic resistance in *Campylobacter* strains poses a serious challenge on the world stage, which the World Health Organization (WHO) has recognized as a serious public health problem [20]. Although there are variations among different strains and different geographic regions, most are resistant to antibiotics such as fluoroquinolones, tetracycline, and erythromycin. The cause of this resistance lies in the indiscriminate use of antibiotics in animal production for decades to control, prevent, and treat infections and enhance animal growth [14,17,19].

*Campylobacter* strains are known for their diversity in terms of ecological niches and physiological characteristics. The ecological adaptability of *Campylobacter* strains contributes to their persistence and prevalence in various environments, emphasizing the importance of a comprehensive approach to studying and managing Campylobacter infections [11,21,22]. Understanding the ecology of *Campylobacter* in non-systemic backyard chickens and monitoring antibiotic resistance is crucial for implementing effective control measures to ensure food safety and mitigate the risk of the emergence and spread of antibiotic-resistant strains in both animal and human populations [3].

Many studies have been conducted to characterize the presence of *Campylobacter* in poultry. These studies aim to understand the prevalence, distribution, and factors influencing the contamination of poultry products in industrial areas and poultry slaughterhouses or retail establishments [23,24,25,26]. However, none of these studies performed an in-depth mapping of the dynamic ecology of *Campylobacter* strains isolated from chicken tissues in rural households, using different nutrient substrates and growth conditions, in order to identify emerging strains found in very small micro-communities. We previously isolated eighteen species of the *Campylobacter* genus from various types of tissues of free-grazing chickens in rural environment, an impressive abundance that suggests that this genus can survive in more environmental niches than has been thought [26]. In the present study, we continued the in-depth analysis of *Campylobacter* isolates obtained from quantitative analysis (bacterial colony counts), also determining the antimicrobial susceptibility profile and the presence of genetic markers of resistance.

## 2. Materials and Methods

The present research article is an extended paper based on the previous one, and thus the details of research areas, methods used for sampling on farms, and carcass management are thoroughly described, point by point, in our previous published study [26]. Briefly, two main elements of our experimental design are important to restate: (a) the selection criteria of rural households and (b) the type of tissue section taken from each carcass (Table 1; Supplementary Material File S1). More specifically:

(a) The following four criteria formed a total of 15 sampling study groups. These criteria were determined solely by deductive reasoning since other similar studies do not exist. The size of the flock, the co-existence of other poultry species, or of small ruminants and/or pigs in the same household and the feeding practices could be parameters able to shape the ecology of *Campylobacter* species by favoring (or not) various niches.
  (i) The size of the flock: up to 15 birds (*Gallus domesticus*), 16–40 birds, and 41 up to 60 birds.
  (ii) The presence or not in the same household of other poultry species like turkeys, ducks, etc.
  (ii) The presence or not in the same household of small ruminants (sheep and goats) and pigs.
  (iv) The administration of households’ leftovers of plant origin (potatoes, tomatoes, etc.) or the administration of industrial-grade concentrated feeds (corn, barley, etc.).

Note of Clarification: For every one of the 15 groups, 20 households were arbitrary selected from the registration list of the local Veterinary Directorate. The number of sampled birds per household, however, was different depending on the flock size: one bird, two birds, and three birds for small, medium, and large flock sizes, respectively.

(b) The following tissue sections were obtained from each bird:
  (i) Approximately 30 g of chicken skin.
  (ii) A total of 100 g of pectoralis muscle.
  (iii) Five swabs from the visceral cavity and the matching liver.

### 2.1. Microbiological Analyses for Isolation of Presumptive Campylobacter spp. Isolates

As already mentioned in our previous study [26], two parallel analytical procedures were followed: a quantitative (enumeration of total *Campylobacter* colony counts) and a qualitative (presence/absence) analysis of the samples. The sub-sections below describe the quantitative method of analysis of the samples. Strains isolated by this method were used to study the ecology of *Campylobacter* species and explore their antibiotic resistance profiles.

#### 2.1.1. Quantitative Analysis

**Figure 1:**
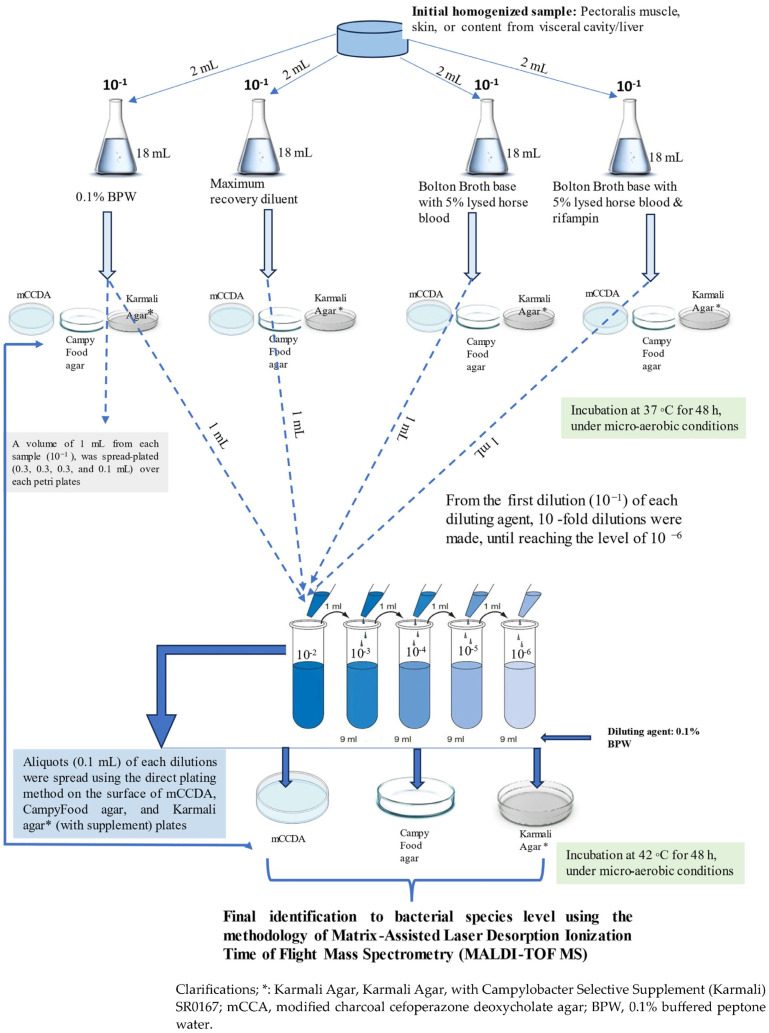
Schematic model, step by step of the experimental procedure for the quantitative analysis of the samples/tissues.

In order to investigate and quantify the presence of *Campylobacter* isolates, each sample was homogenized and then subjected to various technical assays, as illustrated in Figure 1. The main elements that should be highlighted are as follows: the use of four different diluent agents, the use of three different nutrient media, and incubation at two different temperatures at 37 °C or 42 °C under micro-aerobic conditions. The three-nutrient media/procedures were utilized in a complementary manner to maximize the chances of isolating *Campylobacter* strains, in the sense that strains elusive to one medium/procedure could be identified using another method. All isolates were stored in brain heart infusion broth (BHIB) with 50% glycerol at −80 °C until further investigation. *C. jejuni* ATCC 33560 and *C. coli* ATCC 33559 were used in each assay as positive controls.

#### 2.1.2. Species Identification

Isolates that were presumptively characterized as *Campylobacter* spp. were further analyzed using the methodology of matrix-assisted laser desorption/ionization time-of-flight mass spectrometry (MALDI-TOF MS; Bruker Biotyper Microflex; Bremen, Germany) in order to identify the species of each isolate, as described in our previously published study (Figure 1) [26,27,28].

### 2.2. Screening the Antibiotic Susceptibility Pattern of Campylobacter Isolates

A total of 215 *Campylobacter* isolates, belonging to a 17 *Campylobacter* species, were examined in order to evaluate, phenotypically, their antibiotic-resistant profile and the occurrence of antimicrobial resistance-encoded genes. The cryopreserved *Campylobacter* isolates were cultured onto modified charcoal cefoperazone deoxycholate agar plates (mCCDA) (Oxoid, Basingstoke, UK) with the selective supplement SR0155, and the plates were incubated at 42 °C for 48 h under micro-aerobic conditions. In a rectangular 2.5 L capacity container and in order to achieve microaerophilic conditions (5% O_2_, 10% CO_2_, and 85% N_2_), sachets of CampyGen (Oxoid) were introduced. A few drops of glycerol were added to avoid excess moisture, which—if not avoided—could lead to undesirable effects.

#### 2.2.1. Phenotypic Characterization of Antibiotic-Resistant Profile

In the present study, all *Campylobacter* isolates were evaluated for their susceptibility to fourteen (14) antimicrobial agents using the standard Kirby–Bauer disk diffusion method, according to Clinical and Laboratory Standards Institute (CLSI; formerly NCCLS) guidelines [29,30] and three (3) agents via the minimum inhibitory concentrations (MICs), which will be mentioned in the next section. The following antibiotics in paper-disk form from BBL-DIFCO Microbiology, Becton, Dickinson, and Company, U.S.A. were used: nalidixic acid (ΝA), ciprofloxacin (CIP), ampicillin (AΜP), amoxicillin–clavulanic acid (AΜC), erythromycin (ΕRY), tetracycline (ΤΕR), gentamicin (GEN), chloramphenicol (CHL), streptomycin (STM), trimethoprim/sulfamethoxazole (SUT), cephalothin (CFL), cefuroxime (CFU), cefotaxime (CFT), and cefepime (CFE). The reference strain, *E. coli* ATCC 25922, was included in each assay as a quality control. All the antimicrobial susceptibility assays were repeated in triplicate. In Supplementary Material File S2, the concentration in micrograms of each antibiotic substance/paper disk, their abbreviations are shown, as well as the interpretive cut-off values/criteria for inhibition zone diameter size [30,31,32]. The measured inhibition zones of the studied strains were interpreted according to the guidelines and recommendations given by the European Committee on Antimicrobial Susceptibility Testing, EUCAST [31], and in the absence of data, the CLSI guidelines were used [30,32] for Enterobacterales. The multiple antibiotic resistance (MAR) index was calculated using the following formula: a/b, where “a” represents the number of antibiotics to which a particular strain was resistant, and “b” represents the total number of antibiotics that were tested.

The weighted MAR index was calculated by the following formula:

Weighted MAR = Σ (n_i_ MAR_i_)/Σn

where n_i_ is the number of strains of an isolated *Campylobacter* species in an epidemiological group with the same MAR index, MAR_i_ is the multiple antibiotic resistance index of these strains, and n represents the total of *Campylobacter* strains in that group.

#### 2.2.2. Inhibition of the Efflux Pump Factor and Its Effect on the Observed Resistance

To evaluate the impact of efflux pump inhibition on the resistance of Campylobacter isolates, minimum inhibitory concentration (MIC) values for ampicillin, tetracycline, and ciprofloxacin (Oxoid), as well as bile salts (50% sodium cholate and 50% sodium deoxycholate) (Merck), were determined using the broth microdilution method [29] in the presence and absence of carbonyl cyanide 3-chlorophenylhydrazone (CCCP), a well-known efflux pump inhibitor [33]. The antibiotics, representing different classes, were selected based on the phenotypical resistance profile. CCCP is an efflux pump inhibitor that achieves inhibition by uncoupling oxidative phosphorylation and disrupting the proton gradient in the inner membrane, thus inhibiting the rate of ATP synthesis [34]. The antibiotic compounds were serially diluted in Mueller–Hinton (MH) broth in 10 Falcon tubes of 15 mL, obtaining the following concentrations: 512 μg/mL, 256 μg/mL, 128 μg/mL, 64 μg/mL, 32 μg/mL, 16 μg/mL, 8 μg/mL, 4 μg/mL, 2 μg/mL, 1 μg/mL, 0.5 μg/mL, 0.25 μg/mL, and 0.125 μg/mL. For the experiment, 96-well microplates were used; 90 μL was of antibiotic dilution, and 10 μL of the final bacterial inoculum of 10^5^ CFU mL^−1^ was added to each well with a final volume of 100 μL. The serial concentrations for bile salts were from 0.05 to 25 mg mL^−1^, and the wells of the microplates were filled with the same solvent and in the same proportion as in the case of the antibiotics. The CCCP was added to a final concentration of 5 mg mL^−1^, which does not have any inhibitory effect on the bacterial growth, as was checked by a preliminary assay. The MIC measurements were performed in triplicate, including positive and negative controls. Incubation of the microtiter plates took place at 42 °C for 48 h under microaerobic conditions. The antibacterial activity was detected by colorimetry after adding 30 μL of a resazurin staining (0.01%) aqueous solution in each well towards the end of the incubation period (at least 4 h before). The MICs were defined as the lowest concentration of an antibacterial where no metabolic activity is observed after the incubation period, and they were determined by visual observation on the basis of change in resazurin staining (living cells as red; dead cells as blue) [35].

#### 2.2.3. Determination of Antibiotic Resistance-Encoded Genes

For all *Campylobacter* isolates, the presence of the following genes was individually assessed in each isolate: *tet*(*O*), *tet*(*A*), *bla*_OxA-61_, the cluster of *cmeA*, *cmeB*, and *cmeC*, and the occurrence of Thr-86 to Ile mutations (C-to-T transition) in the quinolone resistance-determining region (QRDR) of the *gyrA* gene. The above established resistance to three different antibiotic classes: tetracycline, ampicillin, the CmeABC (efflux system) pump system, and quinolones, respectively. DNA extraction was carried out using the DNeasy^®^ UltraClean^®^ Microbial Kit (Qiagen Inc., Toronto, ON, Canada), according to the manufacturer’s instructions. The extracted DNA of each isolate was stored at −20 °C until further investigations, where it was then used as the template DNA in the PCR. The primer sequences and PCR conditions are listed in Table 2. The final amplification reaction volume was 20 µL, containing 2 µL of template DNA preparation from each *Campylobacter* isolate, 0.5 µL of each primer, 10 µL of (1X) QuantiNova SYBR Green PCR Master Mix (Qiagen, Hilden, Germany), and 7 µL of RNase-free water. The final reaction mixture volume was adjusted to 20 μL. Amplification was performed in a CFX96TM Real-Time PCR thermocycler (BioRad, CA, USA) with reaction conditions as suggested by Poudel et al. [36] and Hungaro et al. [35]. Simplex PCR assays for each gene were performed.

### 2.3. Statistical Analysis

Multilinear regression was used to assess the impact of the various parameters on the ecology of the Campylobacter strains as well as the resistance phenotypes and the distribution of resistant genes. Spearman (r) coefficient was used to estimate the relationship between the different variables. Statistical significance was determined for *p* < 0.05.

## 3. Results

In total, 256 *Campylobacter* isolates were recovered at 42 °C and 255 at 37 °C, with similar but not identical species distributions in the different groups, as shown in Table 3 and Table 4. Both methods were very strongly correlated at an overall genus level (R = 0.9946, *p* < 0.001) as well as at the species level (all Spearman’s rank test r > 0.950 and all *p* < 0.0001). Although 17 species were totally isolated, most of the isolates belonged to *C. jejuni* (37.89%) and *C. coli* (25.00%) species, followed by *Campylobacter lari* (8.98%), *Campylobacter fetus* (7.42%), *Campylobacter avium* (6.64%), and other species in smaller numbers (percentages of isolations at 42 °C).

The counts varied between 1.20 log/CFU and 2.40 log/CFU (for both 42 °C and 37 °C methods, as incubation temperature in microbiological analyses). In the cases where bacteria from the same *Campylobacter* species were isolated from more than one tissue in the same bird, the counts from the skin sample were somewhat higher than the ones from pectoral muscle samples, which in turn were slightly higher than the ones from the samples originating from the liver and the visceral cavity, with these differences not being statistically significant.

The overall statistical association (multilinear regression) with the four studied criteria (which formed the 15 epidemiological sampling groups) selected to assess the prevalence of the *Campylobacter* species in populations of rural-reared chickens revealed that, in general, the small size of the flock acted protectively. Conversely, the large size of the flock had an opposite effect, favoring the diverse presence of the *Campylobacter* species (R = 0.954, *p* < 0.001).

For certain species, however, other studied criteria also played an important role in their distribution, as observed in the case of *Campylobacter fetus*, where the presence of small ruminants and/or pigs in the same household as the birds strongly and positively determined its distribution, while the large size of the flock was a restricting factor, like the small size (R = 0.814, *p* = 0.006). The specifics of the ecology of *Campylobacter* species distribution will be further discussed in the next section.

Besides determining the prevalence of the genus *Campylobacter*, the aim of this study was to investigate the resistance of the isolated strains to antibiotics. Table 5 displays the phenotypical resistance to 14 antibiotics, whose modes of action collectively cover the entire spectrum of drugs used in medical and veterinary practice. Beta-lactams numerically gather the majority of resistance, particularly cephalothin (first-generation cephalosporin, 182 resistant strains), cefuroxime (second-generation cephalosporin, 128 resistant strains), and ampicillin (122 resistant strains). Quinolones, represented by ciprofloxacin, followed by 109 resistant strains, and then tetracycline (oxytetracycline group), followed by 101 strains. Obviously, substances with similar chemical structures exhibited comparable distributions, although this was not always the case. For instance, in the case of amoxicillin–clavulanic acid, the observed resistance was minimal, and in the case of cefepime, there was only one resistant strain.

Multiple antibiotic resistance strains (MAR) were abundant (72.07%). Table 6 illustrates the number of antibiotics against which the various *Campylobacter* isolates demonstrated resistance and their ecological distribution across different epidemiological groups. Overall, a multi-resistance profile was constrained by the small size of the flock but was facilitated by the large size of the flock and the presence of small ruminants and/or pigs in the same household (R = 0.933, *p* < 0.001). Table 7 details the impact of each parameter in the studied “epidemiological type” criteria on the emergence of resistance to each tested antibiotic. Table 8 displays the number of studied antibiotics against which the isolated strains of the most prevalent *Campylobacter* species were resistant.

Molecular methods revealed the presence of genetic determinants, potentially encoding resistance against antibiotics. Table 9 shows the number of isolates possessing such genes or mutations. The cmeABC efflux pump complex shows the highest prevalence, followed by the *gyrA*(Thr-86-Ile mutation) and the *bla*_OxA-61_, and finally the *tet*(*A*) and the *tet*(*O*). The small size of the flock reduces the prevalence of these genes or mutations, while the large size increases it. Only in the case of the *tet*(*A*) gene does the rearing of small ruminants by the same household positively affect its prevalence (R values > 0.717). The *bla*_OxA-61_ gene’s distribution is not influenced by any of these parameters. Table 10 shows the number of antimicrobial resistance-encoded genes detected in the *Campylobacter* isolates as distributed across the studied sampling groups. An interesting observation is that only two isolates possessed all seven studied genes/mutations, while only two isolates possessed none. Once again, the small size of the flock reduces the circulation of antimicrobial resistance-encoded genes, while the opposite effect is observed in the large-sized flocks (R = 0.941, *p* < 0.001).

The MIC challenge of the strains revealed that the inhibition of the CmeABC efflux pump induced by the addition of CCCP did not affect the MIC values in the same way. The ratio of strains whose MIC values were reduced (usually with a reduction factor of 2–4) to the ones whose MIC values remained unaffected varied considerably among the three antibiotics tested (Table 11). Ampicillin’s MIC values were affected in 29.76% of the strains, while tetracyclin’s MIC values were affected in 39.53% of the strains. In the case of ciprofloxacin, however, the majority of the strains’ MIC values (58.14%) were reduced. The effect of the efflux pump system as a mechanism for survival of the *Campylobacter* isolates is shown in Table 11, where the addition of CCCP reduced the MIC values of certain isolates.

Table 12 shows the Campylobacter isolates whose bile salt MIC values were not reduced by the addition of CCCP. These strains represent 24.18% of the total strains.

The weighted MAR index varied in the different sampling groups as well as among isolated species (Table 13). For *C. coli* and *C. jejuni*, the values were strongly positively correlated with the large size of the flock and with the presence of small ruminants and/or pigs (R = 0.847 and R = 0.832, respectively, *p* < 0.001). The prevalence of the genetic determinants of resistance was not affected in the same way by the studied epidemiological criteria. The *bla*_OxA-61_ gene appears unaffected, while all other studied genes/mutation are affected by some of the studied criteria (Table 14).

## 4. Discussion

The backyard-reared chicken is perhaps the most universal dish on the planet since it is present in one form or another in all cultures and on every continent. It is a cheap, tasty, and easy-to-grow source of protein. Yet, most of the scientific research focuses on the quality of the carcasses of industrial broilers. The obvious reason for this discrimination is the fact that the poultry industry, besides its controlled conditions and logistics, is rapidly expanding and is already feeding billions of urban people. On the other hand, the harsh reality that still many—if not most—of the inhabitants of the planet live in rural households should not be neglected. Hence, although no data are available on the population of backyard chickens, their consumed quantities should be at a comparable level to those of the industrial broilers. The backyard hygiene practices concerning the slaughter, and the defeathering, the evisceration is usually compromised. It follows that the risk of *Campylobacter* infections is not a matter to be overlooked.

In our previous research, we studied the prevalence of *Campylobacter* species isolated by both qualitative and quantitative methods and discussed thoroughly the biodiversity of the isolates [26]. We postulated that besides *C. jejuni* and *C. coli*, which are species known to be present in chickens, most of the other isolates could be of either environmental origin, animal origin, or anthropogenic cross-infections [26]. For example, *C. fetus* has been isolated from cattle and sheep [45], *Campylobacter cuniculorum* from rabbits [46], and *Campylobacter upsaliensis* from dogs [47], while *C. rectus* is a member of the human oral flora [48]. In this research, the ecology as well as the resistance to antibiotics of the strains isolated by the quantitative method were studied. The size of the flock was the major factor affecting the prevalence of *Campylobacter* at a genus level, regardless of the temperature of isolation (37 °C or 42 °C). The frequency of isolation decreased in small-sized flocks while increasing in large-sized flocks (R > 0.954, *p* < 0.001.

At the species level, however, the picture was more complicated. The small size of the flock strongly reduced (R > 0.784, *p* < 0.001) the prevalence of *C. fetus* and *C. coli*. The large size of the flock significantly increased the prevalence of *C. jejuni*, *C. coli*, *C. lari*, and *Campylobacter hepaticus* while reducing the presence of *C. fetus* (R > 0.713, *p* < 0.001), affecting thus five out of the six most prevalent species of our study. The presence of small ruminants or pigs in the same household was the second parameter, in descending order of importance. It affected positively the occurrence of *C. jejuni* and *C. fetus* and negatively the occurrence of *C. avium* and *C. hepaticus* (R > 0.730, *p* < 0.001). The fact that backyard chickens are in free contact with pets, pests, and farm animals increases their risk for zoonotic Campylobacter and Salmonella [49]. The size of the flock—in most cases—either grants or restricts the opportunity for a species to thrive. With some exceptions, which have already been mentioned, it seems that the large size favors abundance and diversity. Nather et al. (2009) came to a similar conclusion by studying free-grazing chicken [50].

*Campylobacter* species is a major concern for public health not only because it is the leading cause of bacterial gastroenteritis [3,51,52] but also because, according to a WHO report, it is considered one of the 12 bacterial species that pose a serious threat to human health due to antibiotic resistance [53]. Our findings (Table 6) support this thesis since only 13 strains out of 215 tested (6.05%) were found to be sensitive to all tested antibacterial substances, leaving the rest 202 strains resistant to at least one substance, while MAR strains represented 71.63% (154/215) of the total. A similar pattern of high prevalence of multi-resistant strains of *Campylobacter* spp. has been reported by many researchers, e.g., Obaidat and Alshaifat, 2023 [54], from raw sheep and goat milk; Bai et al., 2021 [55], from broiler carcasses in slaughterhouses; Montgomery et al., 2018 [56], from puppies and humans; and Ibrahim et al., 2018 [57], from retail chicken carcasses.

In the present study, the multi-resistance effect was found to be species-specific and dependent on the isolation frequency as well as the ecological niche (as represented by the studied epidemiological “type” group). As Table 8 depicts, *C. jejuni* strains, for example, showed an increasing multi-resistance profile as their prevalence increased, which in turn increased in certain epidemiological groups. However, this was not the case for *C. lari* isolates, where the number of antibiotics against which they were resistant was higher in the three isolates in epidemiological group nine than in the four isolates that emerged in epidemiological group twelve. It seems that the species-specific effect is stronger than the other parameters.

The reported diversity of sample tissues and territories from which MAR *Campylobacter* spp. Has been isolated indicates that these species easily acquire genetic resistance factors [58]. Indeed, in the current study, seven genes—coding resistance to antibacterial substances—were detected in the *Campylobacter* isolates at increased prevalence rates (Table 9). The *cmeA*, *cmeB*, and *cmeC* genes were the most prevalent, implying that the CmeABC efflux pump acts as a major—though not the only—defense mechanism in this species. The addition of CCCP, an inhibitor of the CmeABC efflux pump, did not affect in the same way the resistance—measured in MIC values reduction—against three antibacterials belonging to three different classes (Table 11). The b-lactam resistance depended more on this efflux pump than on the *bla*_OxA-61_ gene since 70.23% of the strains’ MIC values of ampicillin were reduced when CCCP was added. The *bla*_OxA-61_ gene was detected in 97 strains (45.11%), but since it does not encode any efflux pump mechanism, the addition of CCCP should not affect the MIC values. The majority of the strains (*n* = 64) retained their initial MIC values, but almost one-third of them (*n* = 33) showed reduced MIC values, possibly due to the coexistence of cmeABC group genes. The *tet* − genes (*tet*(*A*) and *tet*(*O*)), were detected in 34.41% of the strains and their expression was affected by the addition of CCCP, as shown by the ratio of the strains with reduced tetracycline MIC values (60.46%). *tet*(*A*) and *tet*(*B*) represent efflux pump systems conferring resistance to the class of tetracyclines and are inhibited by the CCCP [59]. The MIC values of ciprofloxacin were the least affected by the addition of CCCP with respect to the other two substances, implying that the efflux pump effect was limited. Hence, the observed resistance to quinolones in this study should be attributed to the presence of the Thr-86 to Ile mutations in the quinolone resistance-determining region (QRDR) of the *gyrA* gene. Indeed, the *gyrA* gene in Gram-negative bacteria codes the enzyme gyrase, which catalyzes the formation of the print of the bacterial circular DNA. Quinolones’ action targets this enzyme, thus inhibiting the replication of the DNA and killing the bacterial cell.

It is interesting that the distributions of all genes/mutations encoding antibiotic resistance were found in positive relation to each other in their distribution in the 15 epidemiological groups (Spearman’s r > 0.721, *p* < 0.001) except for *bla*_OxA-61_. This finding implies that perhaps these genes were co-transferred via mobile elements, which is in accordance with the findings of other authors [60]. Table 14 shows how the occurrence of these genetic elements was affected by the four created ecological criteria parameters. It is obvious that the *bla*_OxA-61_ gene could be circulating independently with respect to the other genes, which in turn were affected to a greater or lesser extent by these parameters. The small size of the flock restricted the transfer of the genes, while the large size had the opposite effect. The presence of other species of poultry affected only the prevalence of *cmeB*, while the presence of small ruminants and/or pigs affected the prevalence of *tetA* and *cmeB*.

By comparing Table 5, Table 7 and Table 14, it can be deduced that a significant portion of the observed resistance lacks a genetic background. The *bla*_OxA-61_ gene’s distribution moderately to strongly correlates with phenotypical resistance to ampicillin and amoxicillin–clavulanic acid (Spearman’s r = 0.571, *p* < 0.05, and r = 0.634, *p* < 0.05, respectively) but not with cephalosporins (*p* > 0.05), despite all belonging to the same group (beta-lactam antibacterials). This finding suggests that resistance to the first two substances is likely extensively related to a genetic determinant. On the other hand, the relationship between the distribution of the mutation in *gyrA* (Thr-86 to Ile mutations) and the phenotypical resistance to ciprofloxacin (quinolones) was very strong (r = 0.822, *p* < 0.0001), indicating that the observed resistance originated from this genetic determinant.

In the *Campylobacter* species, nine different efflux pump systems have been identified [61], but not all of them are sensitive to the CCCP action, and this is perhaps the reason for the remaining resistance as well as the relatively small reduction in the MIC values. The latter, in the current study and for all three antibiotics, were reduced by a factor 2–4, while in the literature, a reduction in MIC values reported is of much higher order [59].

Bile salts are known to disrupt the bacterial membranes, alter the structure of proteins, and form chelating complexes with iron and calcium ions; hence, they possess antibacterial properties [62,63]. The CmeABC efflux pump operates on a variety of substrates, bile salts being one of them. These efflux pumps are necessary for any microorganism to survive the bile acids in the intestine of an animal [64,65]. The addition of CCCP to our experiment affected most of the *Campylobacter* strains’ (75.81%) MIC values for bile salts. Lin et al. (2005) report an increase in cmeABC expression in the presence of bile salts, an observation consistent with the mechanisms of resistance to these salts. In the present study, CCCP may have inhibited cmeABC activity, which may result in decreased MIC values for bile salts due to the inhibition of bile salt efflux. This finding contradicts Lin et al. (2005), who report that the expression of CmeABC has significantly increased in the presence of bile salts, and this effect was dose- and time-dependent [64]. In general, most of the efflux pumps involved in bacterial resistance and bile salt resistance are non-specific [66].

## 5. Conclusions

- The *Campylobacter* species was found to have an impressive diversity in backyard chickens (17 species, 256 strains). As we extensively discussed in a previous study [26], these strains could be of environmental, anthropogenic or animal origin.
- In general, the size of the flock plays an important role in the prevalence of the various species, with the large size favoring the presence of the microorganisms. A similar effect occurs in the presence of small ruminants. The ecology of *Campylobacter* seems to be species-specific.
- Phenotypical resistance against 14 antibacterial pharmaceutical substances was recorded for most of the strains. Only 13 strains were found to be sensitive to all antibiotics, while 48 strains were resistant to one or two antibacterials. MAR resistance was very high (154 strains out of 215, which is 71.62%). In general, the phenotypes of resistance were also affected by the size of the flock as well as by the presence of small ruminants.
- Genes coding antimicrobial resistance were detected (*bla*_OXA-61_, *tet*(*A*), *tet*(*B*), mutative *gyrA*, *cmeA*, *cmeB*, and *cmeC*) in most of the isolated strains. Apart from the *bla_OxA-61_* which moved independently, the other genes’ prevalence was positively related to each other in their distribution in the epidemiological groups, a finding suggesting the possibility of circulating together in mobile genetic elements. This conclusion is also supported by the finding that the epidemiological criteria affected the prevalence of all other genes except for *bla*_OxA-61_.
- The MIC values were seriously affected when the function of efflux pumps was inhibited by CCCP (including in the case of bile acids), implying that these pumps are an essential mechanism of defense and survival for the *Campylobacter* genus in the presence of antibacterial substances.

## Figures and Tables

**Table 1:** Sampling size and groups formed according to the four “epidemiological type” criteria of the study.

Study Group	Epidemiological Criteria Parameters			
	Size of the Flock (Birds)	Presence of Other Poultry Species	Presence of Small Ruminants and/or Pigs	Feeding Approach: (a) Fed Leftovers or (b) Mainly Concentrates
1 (*n* * = 20)	3–15	no	No	(a)
2 (*n* = 20)	3–15	yes	No	(a)
3 (*n* = 20)	3–15	no	Yes	(a)
4 (*n* = 20)	3–15	yes	Yes	(a)
5 (*n* = 20)	3–15	yes	Yes	(b)
6 (*n* = 40)	16–40	no	No	(a)
7 (*n* = 40)	16–40	yes	No	(a)
8 (*n* = 40)	16–40	no	Yes	(a)
9 (*n* = 40)	16–40	yes	Yes	(a)
10 (*n* = 40)	16–40	yes	Yes	(b)
11 (*n* = 60)	41–60	no	No	(a)
12 (*n* = 60)	41–60	yes	No	(a)
13 (*n* = 60)	41–60	no	Yes	(a)
14 (*n* = 60)	41–60	yes	Yes	(a)
15 (*n* = 60)	41–60	yes	Yes	(b)

*: *n* the number of birds sampled per group. For every 1 of the 15 groups, 20 households were randomly selected from the registration list of the local Veterinary Directorate. The number of sampled birds per household, however, was different depending on the flock size: 1 bird, 2 birds, and 3 birds per household for small, medium, and large flock sizes, respectively.

**Table 2:** PCR primer sequences used for detection of specific antimicrobial resistance-encoded genes in the current study, along with their amplicons’ length.

Target Gene	Primer Sequence (5′ to 3′)	Amplicon Length (bp)	References
*tet*(*O*)	F: GCGTTTTGTTTATGTGCG R: ATGGACAACCCGACAGAAG	559	[37,38]
*tet*(*A*)	F: GTGAAACCCAACATACCCC R: GAAGGCAAGCAGGATGTAG	888	[39]
*bla*_OxA-61_	F: AGAGTATAATACAAGCG R: TAGTGAGTTGTCAAGCC	372	[35,40]
*gyrA* (Thr-86-Ile mutation)	F: TTTTTAGCAAAGATTCTGAT R: CAAAGCATCATAAACTGCAA	265	[36,41]
*cmeA*	F: TGTGCATCAGCTCCTGTGTAA R: ACGGACAAGCTTTGATGGCT	957	[37,42]
*cmeB*	F: GGTACAGATCCTGATCAAGCC R: AGGAATAAGTGTTGCACGGAAATT	820	[42,43]
*cmeC*	F: AGATGAAGCTTTTGTAAATT R: TATAAGCAATTTTATCATTT	500	[42,44]

**Table 3:** Isolated *Campylobacter* species and the numbers of *Campylobacter* isolates (in each species) recovered from rural populations of backyard-reared chicken as distributed across the different studied epidemiological groups (at 42 °C, as incubation temperature in microbiological analyses).

Study Group ^a^	Isolated *Campylobacter* Species																	
	Je ^b^	co	la	fe	av	hp	hl	ur	sp	hy	gr	cc	sh	cu	Mu	re	up	Total
1	-	3	-	-	-		1	-	-	-	-	-	-	-	-	1	1	6
2	2	3	-	-	2	2	-	-	-	-	-	-	-	-	-	-	-	9
3	5	2	-	-	-	-	-	-	1	-	-	-	-	-	1	-	-	9
4	3	1	2	1	-	-	-	-	-	-	-	-	-	-	-	-	-	7
5	3	1	2	1	-	-	-	-	-	-	-	-	-	-	-	-	-	7
6	3	2	-	-	3	1	-	1	-	-	1	1	-	-	-	1	-	13
7	2	4	-	-	3	-	-	-	-	-	1	-	-	1	-	-	-	11
8	4	3	-	5	-	-	-	-	1	1	-	-	-	-	-	-	-	14
9	9	5	3	-	5	-	-	-	-	-	-	-	-	-	-	-	-	22
10	7	7	1	3	-	-	-	-	-	-	-	-	-	-	-	-	-	18
11	-	9	4	-	5	3	-	4	-	-	-	1	1	-	-	-	-	27
12	11	8	4	-	2	2	-	-	-	-	-	-	-	-	-	-	-	27
13	13	5	2	2	-	-	-	-	2	2	-	-	-	-	-	-	-	26
14	18	7	2	2	-	-	-	-	-	1	-	-	-	-	-	-	-	30
15	18	4	3	-	2	2	-	-	-	-	-	-	-	-	-	-	1	30
Total	98	64	23	14	22	10	1	5	4	4	2	2	1	1	1	2	2	256

^a^: The 15 sampling studied groups as shaped according to the four criteria, with their individual recombination’s, as shown in Table 1 and Supplementary Material File S1. ^b^: Je: *C. jejuni*; co: *C.coli*; la: *C. lari*; fe: *C. fetus*; av: *C. avium*; hp: *C. hepaticus*; hl: *C. helveticus*; ur: *C. ureolyticus*; sp: *C. sputorum*; hy: *C. hyointestinalis*; gr: *C. gracilis*; cc: *C. concisus*; sh: *C. showae*; cu: *C. cuniculorum*; mu: *C. mucosalis*; re: *C. rectus*; up: *C. upsaliensis.*

**Table 4:** Isolated *Campylobacter* species and the numbers of *Campylobacter* isolates (in each species) recovered from rural populations of backyard-reared chicken, as distributed across the different studied epidemiological groups (at 37 °C, as incubation temperature in microbiological analyses).

Study Group ^a^	Isolated *Campylobacter* Species																	
	Je ^b^	co	la	fe	av	hp	hl	ur	sp	hy	gr	cc	sh	cu	Mu	re	up	Total
1	-	2	-	-	-		1	-	-	-	-	-	-	-	-	-	1	4
2	1	3	-	-	2	2	-	-	-	-	-	-	-	-	-	-	-	8
3	5	2	-	-	-	-	-	-	1	-	-	-	-	-	1	-	-	9
4	3	1	2	1	-	-	-	-	-	-	-	-	-	-	-	-	-	7
5	3	1	2	1	-	-	-	-	-	-	-	-	-	-	-	-	-	7
6	3	2	-	-	4	1	-	2	-	-	1	1	-	-	-	1	-	15
7	2	4	1	-	3	-	-	-	-	-	1	-	-	1	-	-	-	12
8	4	3	-	5	-	-	-	-	1	1	-	-	-	-	-	-	-	14
9	9	5	3	-	5	-	-	-	-	-	-	-	-	-	-	-	-	22
10	7	7	1	3	-	-	-	-	-	-	-	-	-	-	-	-	-	18
11	-	9	4	-	5	3	-	4	-	-	-	1	1	-	-	-	-	27
12	11	8	4	-	2	2	-	-	-	-	-	-	-	-	-	-	-	27
13	13	5	2	2	-	-	-	-	2	2	-	-	-	-	-	-	-	26
14	18	7	2	2	-	-	-	-	-	1	-	-	-	-	-	-	-	30
15	17	4	3	-	2	2	-	-	-	-	-	-	-	-	-	-	1	29
Total	97	63	24	14	23	10	1	6	3	4	2	2	1	1	1	1	2	255

^a, b^: Abbreviations follow exactly the same pattern, as described in Table 3.

**Table 5:** In numerical terms the phenotypically resistant strains in studied epidemiological group against 14 different antibiotics agents.

Study Group ^a^	AMC ^b^	AMP	CIP	NAL	CHL	ERY	TER	GEN	STM	SUT	CFL	CFU	CFT	CFE
1	0	2	1	0	0	0	0	0	0	0	5	4	0	0
2	1	3	1	0	0	0	0	0	0	0	6	7	0	0
3	0	6	2	0	0	0	1	1	0	1	6	0	0	0
4	2	9	3	4	0	1	1	0	0	3	9	5	3	0
5	1	1	0	3	0	0	2	0	0	0	6	5	3	0
6	3	8	0	0	0	0	0	0	0	0	9	8	1	0
7	1	6	1	2	0	0	2	0	0	0	2	1	0	0
8	1	6	4	1	0	0	5	0	1	2	8	5	1	0
9	2	13	11	6	1	5	13	3	4	11	16	12	2	0
10	5	11	7	6	0	4	9	0	1	10	14	8	2	0
11	1	9	9	0	0	2	9	3	2	15	15	3	1	1
12	0	6	16	1	0	0	14	2	1	19	21	11	2	0
13	2	10	15	5	1	2	13	1	13	16	17	16	9	0
14	2	15	21	1	1	1	17	0	9	18	23	23	5	0
15	5	17	18	3	0	5	15	0	12	15	25	20	13	0
Total	26	122	109	32	3	20	101	10	43	110	182	128	42	1

^a^: The 15 sampling studied groups as shaped according to the four criteria, with their individual recombination’s, as shown in Table 1 and Supplementary Material File S1; ^b^: Amoxicillin-clavulanic acid (AΜC); ampicillin (AΜP); ciprofloxacin (CIP); nalidixic acid (ΝAL); chloramphenicol (CHL); erythromycin (ΕRY); tetracycline (ΤΕR); gentamicin (GEN); streptomycin (STM); trimethoprim/sulfamethoxazole (SUT); cephalothin (CFL); cefuroxime (CFU); cefotaxime (CFT); and cefepime (CFE).

**Table 6:** Phenotypically multiple antibiotic resistance strains (MAR) isolates against antibiotics in the studied epidemiological groups.

Study Group ^b^	Number of Antibiotics ^a^													MAR Index ^c^
	0	1	2	3	4	5	6	7	8	9	10	11	Total	
1	-	2	5	-	-	-	-	-	-	-	-	-	7	0
2	-	2	7	-	-	-	-	-	-	-	-	-	9	0
3	1	4	1	1	2	-	-	-	-	-	-	-	9	3
4	-	1	2	1	-	4	2	-	-	-	-	-	10	7
5	-	1	1	1	1	1	1	-	-	-	-	-	6	4
6	2	2	2	5	2	-	-	-	-	-	-	-	13	7
7	5	1	4	2	-	-	-	-	-	-	-	-	12	2
8	4	1	1	1	1	1	2	1	-	-	-	-	12	6
9	-	-	-	-	3	3	4	1	4	1	-	-	16	16
10	-	-	1	3	1	1	2	4	1	1	-	-	14	13
11	1	2	4	5	6	1	1	-	-	-	1	-	21	13
12	-	1	3	3	7	4	2	-	2	-	-	-	22	18
13	-	-	-	-	-	7	1	2	3	-	3	1	17	18
14	-	-	1	-	4	3	8	5	1	1	-	-	23	22
15	-	-	-	1	6	5	2	5	5	1	-	-	25	25
Total	13	17	32	25	33	30	27	18	18	4	4	1	215	154

^a^: Number of antibiotics agents showing phenotypic resistance; ^b^: The 15 sampling study groups as shaped according to the four criteria, with their individual recombination’s, as shown in Table 1 and Supplementary Material File S1; ^c^: Multiple antibiotic resistance (MAR) index, calculated using the following formula: a/b, where “a” represents the number of antibiotics to which a particular strain was resistant, and “b”, where the total number of antibiotics that were tested.

**Table 7:** The effect of the different studied “epidemiological type” criteria parameters on the phenotypic resistance profile of the *Campylobacter* isolates.

Antibiotics Agents	Criteria with Their Individual Recombination’s Shaped the Sampling Study Groups					
	Small Size (the Size of the Flock: Up to 15 Birds)	Medium Size (the Size of the Flock: 15–40 Birds)	Large Size (the Size of the Flock: More than 40 Birds up to 60)	Presence of Other Poultry Species	Presence of Small Ruminants/or and Pigs	Feeding Approach: Mainly Administration of Concentrated Foods
AMC ^a^	(−) ^b^	NA	(+)	NA	NA	(+)
AMP	(−)	NA	NA	NA	(+)	NA
CIP	NA	NA	(+)	NA	(+)	NA
NAL	NA	NA	NA	(+)	NA	NA
ERY	NA	NA	NA	NA	NA	(−)
TER	(−)	NA	(+)	NA	(+)	NA
GEN	NA	NA	(+)	NA	NA	NA
STM	NA	NA	(+)	NA	(+)	NA
SUT	NA	NA	(+)	NA	NA	NA
CFT	NA	NA	(+)	NA	(+)	NA
CFL	NA	NA	(+)	NA	NA	NA
CFU	NA	NA	(+)	(+)	NA	NA

^a^: Amoxicillin–clavulanic acid (AΜC); ampicillin (AΜP); ciprofloxacin (CIP); nalidixic acid (ΝAL); erythromycin (ΕRY); tetracycline (ΤΕR); gentamicin (GEN); streptomycin (STM); trimethoprim/sulfamethoxazole (SUT); cephalothin (CFL); cefuroxime (CFU); cefotaxime (CFT); Chloramphenicol and cefepime are not included due to very small sample size of resistant strains. ^b^: (−) negative effect, reduced occurrence of resistant isolates; (+) positive effect, increased occurrence of resistant isolates; NA: not affected.

**Table 8:** In numerical terms, the phenotypical resistance profile of each isolate of the most prevalent *Campylobacter* species.

Study Group	Isolated *Campylobacter* Species					
	*C. jejuni*	*C. coli*	*C. lari*	*C. avium*	*C. fetus*	*C. hepaticus*
1	- *	2 **,2,2	-	2	-	-
2	2,2	2,2,2	-	2,1	-	2,1
3	2,3,1,1,4	4,0	-	-	-	-
4	6,5,6	5,5	-	-	3,5	-
5	2,3	6	5,4	-	1	-
6	2,3,2	3	-	3,3,3	-	1
7	0,3	2,2,1,2	0	3,0,2	-	-
8	4,2,6,7	3,6,1	-	-	0,5,0	-
9	7,8,6,8,8	6,5,6	9,5,8	5	6,4,4,4	-
10	9,7,6,7,4	7,5,3,3,8	7	-	2,3,7	-
11	-	6,2,4,2,4, 4,4,4	2,4	4,3,2,3	-	1,3,0
12	3,2,5,4,5, 4,6,8,4	1,3,2,4,8	4,4,4,6	2,3	-	5,5
13	11,10,6,7, 5,5,5,6	8,6,10,8,10	5,5	-	5	-
14	7,7,8,7,6,6, 6,6,5,7,2,7	9,6,4,6,5,4	6,5	-	4,4	-
15	9,7,5,4,4,4, 8,7,6,8,5,8,8	7,7,5,8	4,5	5,4	-	6,7

*: no *Campylobacter* species was isolated; **: the number of antibiotic substances against which each isolate was found resistant. Clarification: each number corresponds to one isolate.

**Table 9:** Frequency, expressed as numbers, of isolates possessing genetic determinants genes/mutations for antibiotic resistance, distributed across the studied epidemiological groups.

Study Group ^a^	Antibiotic Resistance-Encoded Genes/Mutations						
	*bla*_OxA-61_	*tet*(*O*)	*tet*(*A*)	*gyrA* (Thr-86-Ile Mutation)	*cmeA*	*cmeB*	*cmeC*
1	1 ^b^	2	3	0	5	3	1
2	6	0	3	0	7	6	3
3	5	0	4	5	8	4	4
4	8	3	5	6	7	5	6
5	1	1	2	4	4	5	3
6	8	1	2	10	8	11	8
7	6	1	4	7	11	9	6
8	5	4	4	7	12	11	6
9	13	7	9	11	15	15	14
10	11	6	7	11	11	14	14
11	11	6	4	8	15	14	12
12	6	10	6	17	21	13	7
13	3	10	6	11	15	19	17
14	6	7	7	18	12	23	24
15	7	16	9	14	18	24	24
Total	97	74	75	129	169	176	149

^a^: The 15 sampling study groups as shaped according to the four criteria, with their individual recombination’s, as shown in Table 1 and Supplementary Material File S1; ^b^: the number of the isolates possessing genetic determinants genes/mutations.

**Table 10:** Isolates distributed across different studied epidemiological groups, harboring genetic elements (genes/mutations) coding resistance to antibiotics (at 42 °C, as incubation temperature in microbiological analyses).

Study Group	Number of Genes/Mutations Coding Resistance to Antibiotics								
	0	1	2	3	4	5	6	7	Total
1	1 *	1	2	1	2				7
2	1	1	3		1	3			9
3		2	2		2	2	1		9
4			1	3	3	2	1		10
5			1	2	3				6
6			2	3	5	3			13
7		3	1	1	2	2	3		12
8			3	2	1	3	3		12
9				2	2	3	8	1	16
10				1	1	6	5	1	14
11			5	2	2	4	4		17
12		1	5	5	3	5	3		22
13			3	3	7	5	2		20
14			2	6	6	8	2		24
15				3	6	10	5		24
Total	2	8	30	34	46	56	37	2	215

*: the number of the isolates possessing genetic determinants genes/mutations.

**Table 11:** The effect of the agent’s carbonyl cyanide 3-chlorophenylhydrazone (CCCP) presence on MIC values of three antibiotics against *Campylobacter* isolates.

Study Group	AMP ^a^		TET		CIP	
	Reduced MIC	Not Affected	Reduced MIC	Not Affected	Reduced MIC	Not Affected
1 (n ^b^ = 7)	6	1	2	5	2	5
2 (*n* = 9)	6	3	9	0	1	8
3 (*n* = 9)	8	1	5	4	5	4
4 (*n* = 10)	10	0	8	2	4	6
5 (*n* = 6)	6	0	5	1	2	4
6 (*n* = 13)	10	3	8	5	1	12
7 (*n* = 12)	12	0	6	6	1	11
8 (*n* = 12)	11	1	9	3	6	6
9 (*n* = 16)	15	1	14	2	11	5
10 (*n* = 14)	10	4	10	4	5	9
11 (*n* = 17)	5	12	10	7	3	14
12 (*n* = 22)	12	10	10	12	11	11
13 (*n* = 20)	13	7	8	12	6	14
14 (*n* = 24)	14	10	13	11	15	9
15 (*n* = 24)	13	11	13	11	17	7
Total (*n* = 215)	151	64	130	85	90	125

^a^: Ampicillin (AΜP); tetracycline (ΤΕR); ciprofloxacin (CIP); ^b^: the number of the studied isolates.

**Table 12:** Frequency of *Campylobacter* isolates whose bile salts MIC values were not affected by the addition of carbonyl cyanide 3-chlorophenylhydrazone (CCCP).

Isolates with Species Specification	*n* (% of the Total 52 Resistant)	% (of the Total Isolates of Each Isolated Species, at 42 °C, as Incubation Temperature in Microbiological Analyses)
*C. jejuni*	22 (42.31%)	22.45%
*C. coli*	10 (19.23%)	15.63%
*C. fetus*	4 (7.69%)	28.57%
*C. avium*	7 (13.46%)	31.81%
*C. lari*	3 (5.77%)	13.04%
*C. mucosalis*	1 (1.92%)	100.00%
*C. concisus*	1 (1.92%)	50.00%
*C. hepaticus*	2 (1.92%)	20.00%
*C. hyointestinalis*	1 (1.92%)	25.00%
*C. sputorum*	1 (1.92%)	25.00%
Total	52	

**Table 13:** Weighted MAR index of the most prevalent *Campylobacter* species as distributed in the studied epidemiological groups.

Study Group	*Campylobacter* Species					
	*C. coli*	*C. jejuni*	*C. lari*	*C. avium*	*C. fetus*	*C. hepaticus*
1	0.071 ^a^	-	-	0.143	-	-
2	0.071	0.143	-	0.107	-	0.107
3	0.143	0.195	-	-	-	-
4	0.357	0.402	-	-	0.286	-
5	0.429	0.178	0.320	-	0.071	-
6	0.214	0.166	-	0.214	-	0.071
7	0.124	0.107	0	0.118	-	-
8	0.238	0.337	-	-	0.118	-
9	0.402	0.525	0.521	0.357	0.320	-
10	0.369	0.469	0.500	-	0.286	-
11	0.266	-	0.214	0.214	-	0.095
12	0.256	0.323	0.320	0.178	-	0.357
13	0.625	0.488	0.357	-	0.357	-
14	0.402	0.408	0.391	-	0.286	-
15	0.479	0.453	0.320	0.320	-	0.462

^a^: The weighted MAR index was calculated by the formula: weighted MAR = Σ (n_i_ MAR_i_)/Σn. Where n_i_ is the number of strains of an isolated *Campylobacter* species with the same MAR in an epidemiological group, MAR_i_ is the multiple antibiotic resistance of these strains, and *n* represents the total of *Campylobacter* strains in that group.

**Table 14:** The correlation between the different studied “epidemiological type” criteria and the presence of resistance genetic elements of the *Campylobacter* isolates.

Antibiotic Resistance-Encoded Genes/Mutations	Criteria with Their Individual Recombinations Shaped the Sampling Study Groups					
	Small Size (the Size of the Flock: Up to 15 Birds)	Medium Size (the Size of the Flock: 15–40 Birds)	Large Size (the Size of the Flock: More than 40 Birds up to 60)	Presence of Other Poultry Species	Presence of Small Ruminants and/or Pigs	Feeding Approach: Mainly Administration of Concentrated Foods
*bla*_OxA-61_	NA ^a^	NA	NA	NA	NA	NA
*tet*(*O*)	NA	NA	(+)	NA	NA	NA
*tet*(*A*)	(−)	NA	NA	NA	(+)	NA
*gyrA*(Thr-86-Ile mutation)	(−)	NA	(+)	NA	NA	NA
*cmeA*	(−)	NA	(+)	NA	NA	NA
*cmeB*	(−)	NA	(+)	(+)	(+)	NA
*cmeC*	(−)	NA	(+)	NA	NA	NA

^a^: NA: not affected; (−) negative effect, reduced occurrence of resistant isolates; (+) positive effect, increased occurrence of resistant isolates.

## Data Availability

Data are contained within the article.

## Supplementary Materials

The following supporting information can be downloaded at: https://www.mdpi.com/article/10.3390/microorganisms12020368/s1. Supplementary Material File S1: Sampling size and groups formed according the four criteria of the study; Supplementary Material File S2: Interpretation criteria /cut-off values used to characterize the antibiotic resistance pattern of Campylobacter isolates. Interpretation values according to EUCAST 2023 and CLSI, 2012 & 2021 for *Enterobacterales* [28,29,30].
