# Supplementary material for: Overview of Ecology and Aspects of Antibiotic Resistance in Campylobacter spp. Isolated from Free-Grazing Chicken Tissues in Rural Households
Source: Microorganisms. 2024 Feb 10;12(2):368. doi: 10.3390/microorganisms12020368 (PMC10892918; doi:10.3390/microorganisms12020368)
Supplement: Supplementary file 1 [file microorganisms-12-00368-s001.zip › File S2.pdf]

Antibiotic agent	Disk code	Disk Concentration	Cut-ff criteria interpretation in diameter of inhibition zone in millimeter (mm)[a]		
			Resistant strain (R)	Intermediate strain (I)	Susceptible strain (S)
Ampicillin	AMP-10	10 µg	≤ 13	14-16	≥ 17
Amoxicillin-clavulanic acid	AMC-10	20/10 µg	≤ 13	14-17	≥ 18
Ciprofloxacin	CIP-5	5 µg	< 26	-	≥ 26
Nalidixic acid	NAL-30	30 µg	≤ 13	14-18	≥ 19
chloramphenicol	CHL-30	30 µg	≤ 12	13-17	≥ 18
Erythromycin (*C. jejuni*)	ERY -15	15 µg	< 20	-	≥ 20
Erythromycin (*C. coli*)	ERY -15	15 µg	< 20	-	≥ 20
Gentamicin	GEN -10	10 µg	≤ 12	13-14	≥ 15
Tetracycline	TER- 30	30 µg	< 30	-	≥ 30
Streptomycin	STM-10	10 µg	≤ 11	13-14	≥ 15
Trimethoprim /sulfamethoxazole	SUT -25	23,75/1,25 µg	≤ 10	11-15	≥ 16
Cephalosporins					
Cephalothin (1th generation)	CFL-30	30 µg	≤ 14	15-17	≥ 18
Cefuroxime (2th generation)	CFU-30	30 µg	≤ 14	15-17	≥ 18
Cefotaxime (3th generation)	CFT-30	30 µg	≤ 22	23-25	≥ 26
Cefepime (4th generation)	CFE-30	30 µg	≤ 14	15-17	≥ 18

S 2. Interpretation criteria /cut-off values. used to characterize the antibiotic resistance pattern of *Campylobacter* isolates. Interpretation values according to EUCAST 2023 & CLSI, 2012 & 2021 for Enterobacterales [28-30].
