# Supplementary figures and images for: Overview of Ecology and Aspects of Antibiotic Resistance in Campylobacter spp. Isolated from Free-Grazing Chicken Tissues in Rural Households
Source: Microorganisms. 2024 Feb 10;12(2):368. doi: 10.3390/microorganisms12020368 (PMC10892918; doi:10.3390/microorganisms12020368)

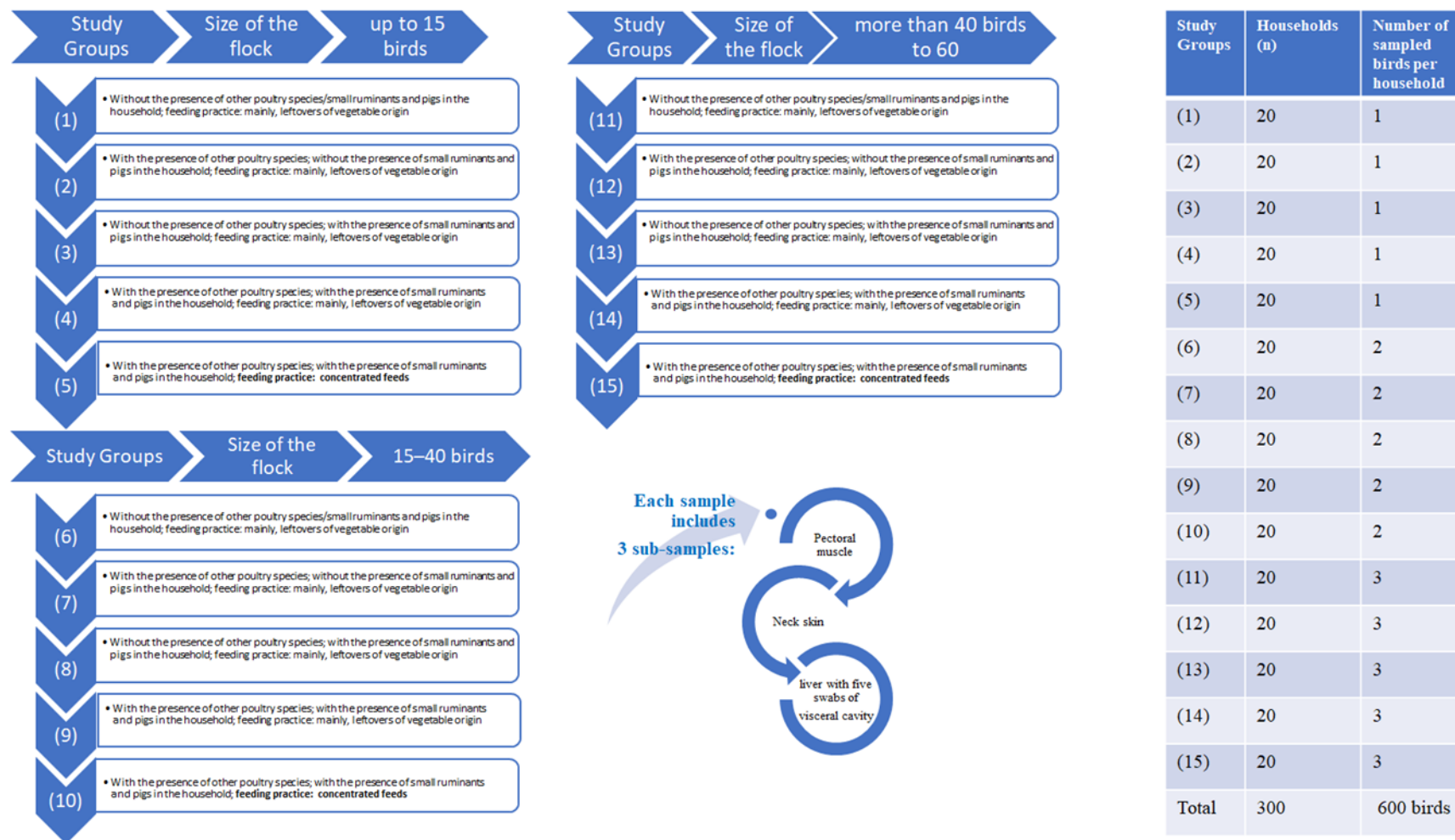

**S 1:** Sampling size and groups formed according the four criteria of the study

Supplement: Supplementary file 1 [file microorganisms-12-00368-s001.zip › File S1.pdf]
